# Strengthening paper health register systems: strategies from case studies in Ethiopia, Ghana, South Africa and Uganda

**DOI:** 10.7189/jogh-06-020303

**Published:** 2016-12

**Authors:** Eva W Westley, Sharon A Greene, Gillian A M Tarr, Tove K Ryman, Sarah Skye Gilbert, Stephen E Hawes

**Affiliations:** 1 University of Washington, Strategic Analysis, Research and Training (START) Center, Seattle, WA, USA; 2 University of Washington, Department of Epidemiology, Seattle, WA, USA; 3 Bill and Melinda Gates Foundation, Vaccine Delivery Team, Seattle, WA, USA

Health information systems (HIS) include a spectrum of data collection tools that support clinical decision–making; facilitate tracking of patients, drug stock, and disease trends; and inform policymaking [1]. As intermediaries between individual patient records and population–level data, health registers occupy a unique space in HIS. Health registers are “a collection of records containing data about aspects of the health of individual persons” [2].

Paper health registers can be books, folders, or forms that include individual–level data for a population. Paper registers are primarily used at the facility level, though they can serve as inputs to higher level reporting. Because they serve health providers, program administrators, and health management decision–makers, registers can sometimes fail to meet all stakeholder identified needs. Studies of paper registers frequently document data quality challenges which compromise efforts to deliver effective care.

Despite the global shift toward digital data collection, there remain low–resource settings that are unable to support the infrastructure required for electronic register systems. For these settings, strengthening paper health register systems can bolster evidence–based decision–making in patient encounters, program planning and policy, and serve as a first step toward improving quality data in HIS as they shift toward electronic systems.

## STRATEGIES FOR IMPROVING PAPER REGISTER SYSTEMS

We developed case studies on innovations in paper health register systems in low–resource settings in Sub–Saharan Africa. The resulting studies were informed by 14 expert interviews (2–4 per study) and 101 documents, including peer–reviewed and non–peer–reviewed literature. Case studies are described in **Table 1**.

**Table 1:** Summary of case studies*

	Ethiopia’s Family Folder	Ghana’s Simplified Register	South Africa’s 3–Tiered strategy	Uganda’s tuberculosis registers
Health domain	Primary care	Primary care	HIV/AIDS	Tuberculosis
Innovation	Collects patient and household–level information in a folder system; services provided at the individual level are tracked by a tally system.	Condenses all primary health registers into five SRs.	Collects standard, reduced list of essential data elements from facilities using paper or electronic systems.	Records and reports data elements for 22 TB indicators using WHO standardized registers.
Data collection	Collected by health extension workers in the community and at health posts.	Collected by frontline health workers in the community and at health posts.	Collected from clinical stationery by data clerks.	Collected by clinical staff, laboratory staff, and frontline health workers.
Data aggregation	Data aggregated at the primary health unit, which encompasses five to ten health posts.	Data aggregated at the district, regional, and national levels.	Data aggregated at the district, regional, and national levels.	Data aggregated at the district, regional, and national levels.
Integration into national systems	As of February 2014, 75% of health posts in the country use the FF.	MoTeCH implemented the SR in four regions.	Established as national standard for HIV programs in December 2010	National standard since 1990; TB/HIV collaborative registers since 2005.
Decision– making	Data used to optimize decision–making at the local level and to prioritize doorstep care.	Data informs patient care decision–making and defaulter tracking.	Data used for regional and national decisions; district and facility–level decision–making is slowly growing	Data used for national decision–making and international monitoring of TB indicators.
Plans for future sustainability (as of 2014)	Scale–up is continuing.	There is not yet a commitment for national adoption of the SR.	All facilities plan to move from paper toward the electronic tiers over the next few years.	TB/HIV collaborative activities will continue to be supported.

ART – Antiretroviral treatment, FF – Family Folder, MoTeCH – Mobile Technology for Community Health, SR – Simplified Register, TB – Tuberculosis, WHO – World Health Organization

*All case studies are publicly available at: http://technet–21.org/en/forums/case–studies–on–paper–immunization–registers.

This commentary synthesizes lessons learned from these case studies, illuminating four successful strategies for optimizing paper health register systems: support local solutions, align with global standards; collect only essential data elements; foster data use and data quality improvement; and invest in strengthening human resources. Within these strategies we identify specific, actionable recommendations that could be applied by policymakers, facility managers, health workers, or others who are interested in strengthening paper health register systems. While these recommendations may be obvious to those who work in HIS, they are not yet well–documented in the literature.

## SUPPORT LOCAL SOLUTIONS, ALIGN WITH GLOBAL STANDARDS

Many of the challenges with registers occur when these systems are designed and implemented by outside stakeholders not aware of the needs and constraints of frontline register users or the HIS that are already in place. Externally–led efforts can take away a sense of ownership within the health system, result in duplicate data collection, and often increase system fatigue. Frontline health workers may not use registers they find unsatisfactory, and local health authorities may not endorse registers that fail to meet their needs. These case studies demonstrate that register systems inspired by grassroots solutions are often more accepted and more likely to be successfully scaled. Yet global and national standards are essential for consistent measurement and comparability of key health indicators [3,4]. While the data points included in registers should be aligned with global standards, standard registers *designed* by global organizations may not satisfy local needs. Recommendations include:

• Once a problem with the register is identified, connect with frontline register users who encounter that problem for insight

• Invest in buy–in meetings during planning and implementation to bring multiple stakeholders together

• As the register matures, hold periodic stakeholder workshops to sustain support at all levels

## COLLECT ONLY ESSENTIAL DATA ELEMENTS

Data proliferation is a challenge in all HIS but is magnified in paper registers. Efforts to improve efficiency should be undertaken with attention to the register’s purpose and the broader HIS. An important first step in register design is to explicitly determine whether the register needs to inform clinical decision–making, reporting, or both. While stakeholders may generally accept that non–essential data elements should be trimmed, determining how to judge an element as non–essential requires compromise and can be a major challenge. For some health domains, international guidelines stipulate a minimum data set that can be used as a starting point. Integrating vertical health programs and their registers can consolidate the data points collected in a given register. Recommendations include:

• Consider using registers for either patient care or reporting needs (not both) if their dual purpose is detracting from data quality and use.

• Assess which data elements must be reported; define an essential data set.

• Look to internationally agreed upon case definitions and indicators to design a core set of data elements.

• Design official register systems or updates that complement each other for linked areas of care.

• Minimize indicator duplication across health domains.

• Optimize reporting mechanisms, not just content within reports.

• Use an alternative to traditional registers to link individual to aggregate data.

**Figure:**
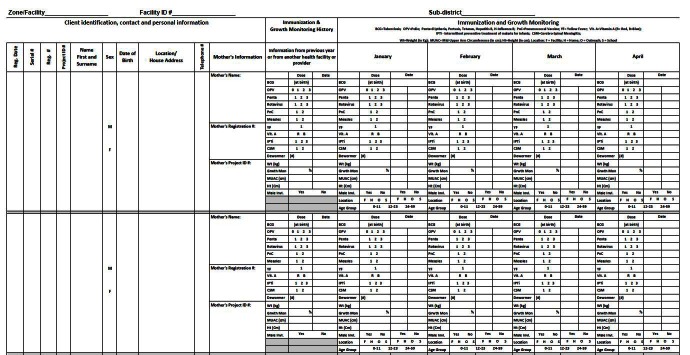
Image: Page from the Ghana Simplified Health Register. Courtesy of the Upper East Regional Health Directorate, Ghana Health Service and the Heilbrunn Department of Population and Family Health, Columbia

## FOSTER DATA USE AND DATA QUALITY IMPROVEMENT

The need to improve data quality was repeated by experts across all four case studies and is of central importance in strengthening register systems. Commitment to utilizing data for evidence–based decision–making is essential at all levels of a register system. When data are of high quality, it is more likely to be used by stakeholders at every level, and when data are considered to be useful it may be collected and aggregated more carefully. Designing registers to support flexible workflows may improve service delivery, register use, and data quality [5]. Efficient guidelines, trainings, and monitoring systems support the correct use of registers. Recommendations include:

• Format registers to support and inform patient care.

• Consider service delivery patterns, including location of service delivery, in register design.

• Allow for variations in register use to support workflows.

• Skip or abbreviate historical data capture for certain types of patients.

• Field–test the register to understand how the full product will be used.

• Include instructions for data collection and reporting on the register itself.

• Design an internal audit system to standardize data quality monitoring.

• Collect register usability data from frontline register users alongside other ongoing monitoring and evaluation efforts.

## INVEST IN STRENGTHENING HUMAN RESOURCES

People who initiated and sustained improvements within these four case studies had various combinations of passion for data, willingness to mentor, and creativity to think differently about register design. Political support at all levels improves the implementation process and contributes to the sustainability of the register system. Key informants across all four case studies identified human resource constraints as a major challenge to the implementation of health registers. These challenges include both lack of personnel and lack of proper training. Human resource interventions should facilitate engagement with register systems. Recommendations include:

• Use peer–to–peer training models

• Enlist influential public figures for training activities to increase worker buy–in

• Recruit key “change agents” to leadership positions within the register system, and encourage their professional growth

• Design staff positions that can easily task–shift as the register system matures

• Relieve the burden on health providers by allocating activities to data clerks

• Ensure that there is dedicated staff time to support the register system at the district or sub–district level

• Implement a supportive supervision model at the district– and facility–level to encourage decision–making with data from the registers

## LIMITATIONS

Efforts were made to include experiences from different geographies, health domains, and stakeholders to best capture the common strengths and challenges faced by paper health registers. The recommendations revealed through these cases are influenced by the particular case studies that were selected and the sources that were most accessible to the researchers. Conclusions may not be generalizable to other contexts.

## THE WAY FORWARD

Paper health registers are important tools in HIS and will continue to occupy a critical role in health service provision, administration, and reporting in many low–resource settings. However, implementing changes to these systems requires commitment of time and resources, and must be approached strategically to avoid system fatigue. Thus, it is important to consider adjustments and updates to multiple aspects of the system. Additional costing studies or operational research could identify efficiencies and reallocate resources toward the most promising solutions. Documenting and sharing lessons learned in other existing HIS can provide additional knowledge to continue to improve these systems.

These cases reveal that there are numerous factors outside of a register’s physical attributes that can be addressed to strengthen register systems, including innovative human resource models, policymaking, and implementation strategies. Many stakeholders—including funders, policymakers, public health officials, and health providers—can be a part of strengthening paper register systems to support accurate reporting, evidence–based decision–making, and improved patient care.

## References

[1] World Health Organization. Everybody's business: strengthening health systems to improve health outcomes: WHO’s Framework for Action. Geneva: WHO Press, World Health Organization, 2007. p 1–56.

[2] Australian Institute for Health and Welfare. Minimum guidelines for health registers for statistical and research purposes. National Health Information Management Group & Australian Institute for Health and Welfare (AIHW). Cat. no. AIHW 9792. Canberra: AIHW, 2001.

[3] Alkraiji AI, Jackson TW, Murray I (2012). The role of health data standards in developing countries.. J Health Inform Dev Ctries..

[4] Mutale W, Chintu N, Amoroso C, Awoonor–Williams K, Phillips J, Baynes C (2013). Improving health information systems for decision making across five sub–Saharan African countries: implementation strategies from the African Health Initiative.. BMC Health Serv Res.

[5] Mobile technology for community health in Ghana: what it is and what Grameen Foundation has learned so far. Grameen Foundation 2012 P1–155. Available at: http://www.grameenfoundation.org/resource/motech-lessons-learned. Accessed: 1 May 2014.

